# Interventions to achieve environmentally sustainable operating theatres: an umbrella systematic review using the behaviour change wheel

**DOI:** 10.1097/JS9.0000000000001951

**Published:** 2024-08-02

**Authors:** Aws Almukhtar, Carys Batcup, Miranda Bowman, Jasmine Winter Beatty, Daniel Leff, Pelin Demirel, Gaby Judah, Talya Porat

**Affiliations:** a Department of General Surgery, Imperial College Healthcare NHS Trust, St Mary’s Hospital; b Department of Surgery and Cancer, Imperial College London, St Mary’s Hospital, 10th Floor Queen Elizabeth Queen Mother Building; c Dyson School of Design Engineering, Imperial College London; d Department of Breast Surgery, Imperial College Healthcare NHS Trust, Charing Cross Hospital, London, UK

**Keywords:** behaviour change, carbon footprint, environment, interventions, surgery, sustainability, umbrella review

## Abstract

**Introduction:**

The healthcare sector is a major contributor to the climate crisis and operating theatres (OTs) are one of the highest sources of emissions. To inform emissions reduction, this study aimed to (i) compare the outcomes of interventions targeting sustainable behaviours in OTs using the Triple Bottom Line framework, (ii) categorise the intervention strategies using the five Rs (reduce, recycle, reuse, refuse, and renew) of circular economy, and (iii) examine intervention functions (IFs) using the Behaviour Change Wheel (BCW).

**Methods:**

Medline, Embase, PsychInfo, Scopus, and Web of Science databases were searched until June 2023 using the concepts: sustainability and surgery. The review was conducted in line with the Cochrane and Joanna Briggs Institution’s recommendations and was registered on PROSPERO. The results were reported in line with Preferred Reporting Items for Systematic reviews and Meta-Analyses (PRISMA) (Supplemental Digital Content 1, http://links.lww.com/JS9/D210) guidelines.

**Results:**

Sixteen reviews encompassing 43 life-cycle analyses, 30 interventions, 5 IFs, and 9 BCW policy categories were included. 28/30 (93%) interventions successfully led to sustainability improvements; however, the environmental outcomes were not suitable for meaningful comparisons due to their using different metrics and dependence on local factors. The ‘reduce’ strategy was the most prolific and commonly achieved through ‘education’ and/or ‘environmental restructuring’. However, single-session educational interventions were ineffective. Improving recycling relied on ‘environmental restructuring’. More intensive strategies such as ‘reuse’ require multiple intervention functions to achieve, either through a sustainability committee or through an intervention package.

**Conclusion:**

Policymakers must examine interventions within the local context. Comparing the outcomes of different interventions is difficult and could potentially be misleading, highlighting the need for a tool integrating diverse outcomes and contextual factors. ‘Reduce’ strategy guarantees environmental and financial savings, and can be achieved through ‘Education’ and/or ‘environmental restructuring’.

## Introduction

Highlights

- Comparing the environmental outcomes of different interventions is currently impossible and potentially misleading.
- Policymakers must scrutinise interventions within the context of their local specific healthcare practices, energy sources, waste management systems, and environmental regulations.
- Of the five Rs, the ‘reduce’ strategy is potentially the most impactful and can be achieved through ‘staff education’ and ‘environmental restrictions’.
- Multifaceted-approach interventions to changing behaviour are highly effective and the presence of a dedicated sustainability committee is a key facilitator in influencing behavioural change in surgery.
- There is a pressing need for a tool that can integrate and synthesise diverse outcomes and metrics from different interventions to enable useful comparisons.

In 2009, Costello *et al*.^1^ conducted a comprehensive review of the impacts of climate change on human health and concluded that climate change is the biggest threat to human health in the 21st century. In 2015, the Lancet Commission on Health and Climate Change argued that tackling climate change is the greatest opportunity of this century given the financial and social co-benefits achieved from actions aimed at decarbonising the economy^2^. At COP 21 in Paris later that year, 196 United Nations (UN) delegations signed a declaration and pledged to reduce Greenhouse Gas (GHG) emissions to avoid a global temperature increase of 2°C by the year 2050^3^. As a result, the United States of America (USA), the United Kingdom (UK), and the European Union (EU) have all set targets to be carbon neutral by 2050.

Healthcare is a significant contributor to GHG emissions through various routes such as waste generation^4,5^ and electricity use^6–8^, and is estimated to be responsible for a 4.4% share of the global carbon footprint^9^. In light of this, various health organisations have set targets to reduce the climate footprint of health services. In the UK, the environmental impact of the National Health Service (NHS) is estimated to be between 4 and 5.9% of total national carbon emissions^10^. Consequently, the NHS has set targets to be carbon neutral by the year 2045^11,12^. In October 2020, the NHS England and NHS Improvement board approved a new strategy to tackle climate change mainly through focusing on energy efficiencies and reducing emissions from transport, supply chains, and medicines^13^. The highest cuts in emissions have thus far been as a result of greening energy grids and changing suppliers; however, the report highlights the change in staff behaviour as a result of the COVID-19 pandemic, such the expanded use of Personal Protective Equipment (PPE) and Heating, Ventilation, Air Conditioning (HVAC) systems. This finding stresses the need for targeted interventions on local institutional and staff levels in the coming years.

Operating theatres (OTs) in particular contribute substantially to the health sector’s GHG footprint due to their energy-intensive nature and extensive use of single-use items. A review by Rizan *et al*.^7^ concluded that a single operation generates up to 814 kg carbon dioxide equivalent (CO_2_e), equating to driving 2273 miles in an average petrol car. The evidence supporting targeted and local interventions is characterised by heterogeneity and limitations^14,15^.

A systematic literature review is necessary to comprehensively examine the various interventions. Our scoping review uncovered that existing reviews have often focussed on particular areas of green practices within OTs such as awake surgeries^16^, infection control^17^, or dermatology^16^. However, some of these interventions were missed in the reviews that aimed to have a broad scope^14,18,19^ leading to notable omissions. Given the complex and interacting systems, it would be beneficial to get a broad understanding of all existing interventions tackling sustainability in OTs in order to inform best practice in future. As such, an umbrella approach is recommended^20^ to provide a comprehensive assessment of the available evidence, that will influence the establishment of guidelines^21^.

Evaluating the environmental outcomes of interventions or Life Cycle Assessment (LCA) studies to identify the impactful interventions can be challenging^22^. LCA studies in particular can be myopic with respect to important outcomes such as long-term effects, cost-effectiveness, and scalability of the particular intervention within a given healthcare authority. The Triple Bottom Line (TBL) framework allows policymakers to consider the social, environmental, and economic outcomes of an intervention^23,24^. For example, interventions that result in both financial and environmental benefits could be favoured over ones that result in only one. Despite some of the challenges in healthcare such as the difficulty in weighing financial gains in the context of health^25^, the TBL does not compare or score dimensions, allowing a greater freedom for policymakers to decide on interventions. As such, the NHS England and other health authorities that endorse sustainability efforts^26^, in addition to empirical studies exemplified by Vergunst *et al*.^24^, demonstrate the value of retrospective TBL assessments for healthcare interventions.

For a systematic approach to examine, select, design, implement, and evaluate strategies, the use of frameworks is crucial. The Sustainable Healthcare Coalition (SHC) advocates for the use of the five Rs (Circular Economy Concepts)^27^. In addition, interventions to increase sustainability often involve changing behaviour. Therefore, behavioural science frameworks should be used to understand effective intervention. The Behaviour Change Wheel (BCW) developed by Michie *et al*.^28^ offers a comprehensive and systematic framework for a nuanced understanding of the underlying determinants of behaviour to enable linking the desired behaviour to an intervention^29,30^. It enables the identification of Intervention functions (IFs), policy categories (PCs), and potential levers, providing a roadmap for policymakers to design or select an intervention that results in effective behaviour change^28,31^. The use of the BCW in healthcare has been proven suitable to categorising a wide range of interventions^29,30,32^. This categorisation not only aids in understanding the complexity of interventions, but also streamlines the process of replication on a local level, facilitating adaptability and applicability in various healthcare settings.

This umbrella review aimed to create a thorough, evidence-based repertoire of interventions and their environmental, financial, and social outcomes using the TBL^24^. These were categorised using the five Rs described in the Centre of Healthcare Sustainability’s Green Surgery Report^27^ and analysed using the BCW. With a diverse array of interventions and their associated evidence, policymakers can prioritise and allocate resources strategically, ensuring a targeted focus on the most impactful strategies. Importantly, policymakers can judge interventions not only in terms of their environmental benefits but also their social and economic feasibility. This comprehensive understanding ensures that the chosen interventions align with the institution’s values, priorities, and resource capabilities, leading to more effective and sustainable practices in OTs.

## Methods

### Search strategy

The review methods were established prior to the conduct of the review, and the review was preregistered on PROSPERO, and on the Open Science Framework (OSF) registry. Medical Subject Heading (MeSH) and non-MeSH search terms encompassing ‘Sustainability’ and ‘Surgery’ were devised with the help of a medical librarian and combined using Boolean string logic. A pretested validated search filter by the Scottish Intercollegiate Guidelines Network (SIGN)^33^ was used to identify only reviews published in Ovid Medline, Ovid Embase, Ovid PsychInfo, Web of Science, and Scopus from inception until June 2023. A snowball manual search for references cited in each review was also conducted. Table 1 in the e-supplement (Supplemental Digital Content 2, http://links.lww.com/JS9/D211) illustrates the complete search terms used.

### Selection process

Articles were uploaded onto Covidence (Veritas Health Innovation. Available at www.covidence.org) and screened by two of three reviewers independently. All studies that looked at the environmental impact of interventions (tested or predicted) in surgery that were published in English were included. The full list of inclusion and exclusion criteria are noted in Table 2 in the e-supplement (Supplemental Digital Content 2, http://links.lww.com/JS9/D211). Discrepancies were resolved through discussion and with the involvement of senior authors.

### Data extraction and synthesis

Data extraction for the reviews and the studies included in the reviews was performed by two of three independent reviewers and conflicts were resolved through discussion and involvement of a senior author. Data regarding the reviews were extracted using a standardised data extraction table (Table 4 in the e-supplement, Supplemental Digital Content 2, http://links.lww.com/JS9/D211). A separate data extraction sheet (Table 5 in the e-supplement, Supplemental Digital Content 2, http://links.lww.com/JS9/D211) was used for the studies included in the review, which were divided into tested interventions (i.e. an effort was made to change behaviour or systems to reduce their environmental impact) and predicted calculations (e.g. the estimated impact of using a reusable piece of equipment instead of the currently used single-use version). Deductive analysis using the TBL and the five Rs described above was conducted for all studies. Deductive analysis using the BCW’s IFs and policy categories (PCs) was conducted for intervention studies. Where this information was not reported in the review that included the study, we reverted to the original study to complete the extraction. Table 1 outlines the definition of the terms used.

**Table 1:** Intervention functions, and their definitions as per the BCW. Concepts of the five Rs and their definitions.

BCW		5Rs	
Intervention functions	Definition	Concept	Definition
Education	Increasing knowledge or understanding	Refuse	Refusing traditional practices and adopting more sustainable alternatives
Persuasion	Using communication to induce positive or negative feelings or stimulate action	Reduce	Reducing the consumption of materials and resources
Incentivisation	Creating expectation of reward	Reuse	Extending the life of materials and products through reusing or repurposing
Coercion	Creating expectation of punishment or cost	Renew	Engaging in safe and effective repair or remanufacture of products to extend their life
Training	Imparting skills	Recycle	Initiating or improving recycling practices to close the loop and minimise waste
Restriction	Using rules to reduce the opportunity to engage in the target behaviour (or to increase the target behaviour by reducing the opportunity to engage in competing behaviours)	Modelling	Providing an example for people to aspire to or imitate
Environmental restructuring	Changing the physical or social context	Enablement	Increasing means/reducing barriers to increase capability or opportunity

### Quality assessment

Quality assessment was done using the JBI critical appraisal tool recommended by the Joanna Briggs Institute (JBI) for the conduct of umbrella reviews^34^. This tool interrogates the reviews on 11 criteria to ascertain the reliability and validity of the findings reported. The reviewer can then judge whether to include, exclude, or seek further information from the authors. (Further details are demonstrated in Table 3 in the e-supplement, Supplemental Digital Content 2, http://links.lww.com/JS9/D211).

### Data analysis and reporting

The results of this review were not intended to be combined using a meta-analysis due to the expected heterogeneity. Quantitative data were presented via tables to aid decision-making on an local level and categorised using the TBL^24^ and the five Rs^35^. Qualitative data were analysed according to the BCW IFs and policy categories^28^. A narrative synthesis was employed to summarise, analyse, and report the data. The results were reported according to Preferred Reporting Items for Systematic Reviews and Meta-Analyses (PRISMA) (Supplemental Digital Content 1, http://links.lww.com/JS9/D210)^36^ guidelines, and the conduct of an umbrella review set by the JBI.

## Results

### Study selection

**Figure 1:**
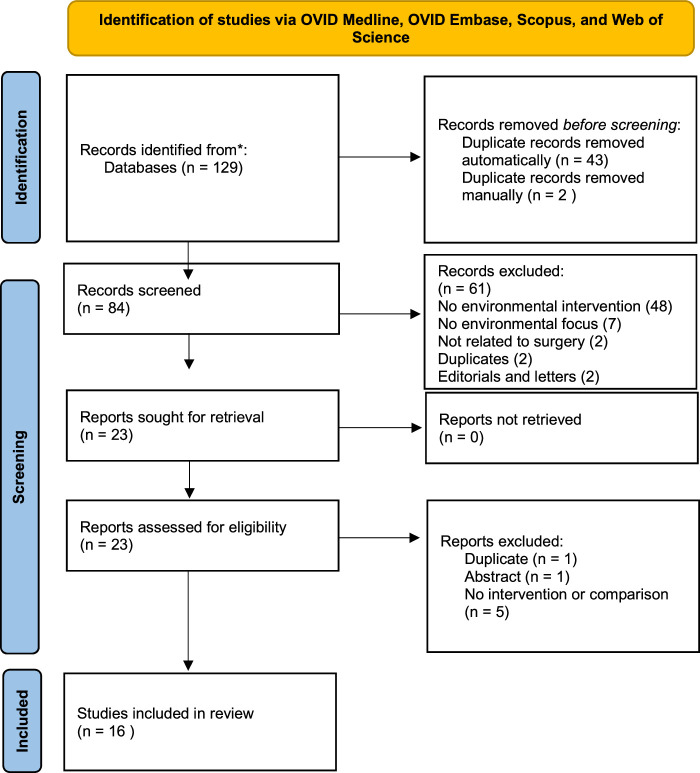
PRISMA^36^ diagram demonstrating the screening and inclusion process. Of the 129 studies, 45 were duplicates, leaving 84 articles which were subjected to title and abstract screening against our inclusion and exclusion criteria. Of these, 23 met our criteria and were subjected to full-text screening. Of these, 7 articles were excluded leaving 16 studies eligible for inclusion in the final review.

The search resulted in 129 reviews. Of these, 16 reviews were included in this review. Figure 1 illustrates the number of studies identified through the database search and screening process, and the reasons for exclusion. Quality assessment was satisfactory for all the included studies. However, three studies^18,37,38^ did not meet the sources of search criterion, and it was not clear how the reported data were used for recommendation in two studies^19,37^. Due to the heterogeneity between studies, most reviews opted not to conduct a quality assessment of the included studies, and rather chose a narrative synthesis. Table 3 in the e-supplement (Supplemental Digital Content 2, http://links.lww.com/JS9/D211) outlines the quality assessment of included studies.

Four studies were from the US^15,16,18,39^, four from the UK^7,14,19,40^, two each from France^38,41^, Australia^42,43^, and Canada^22,44^, one from the Netherlands^17^, and one international^45^. They included systematic, narrative and scoping reviews. Some studies generally investigated the environmental impact of OTs while others had a specific focus (e.g. environmental impact of awake hand surgery). In terms of environmental focus, some reviews investigated carbon footprint, and others focused on wasteful practices. Some chose to include only Life Cycle Analysis (LCA), others only interventions, and others did not describe any specific focus of study methodology. Table 2 summarises the included reviews.

**Table 2:** Summary of included reviews.

Author (Date)	Country	Study design (Dates)	Aim of review	Search strategy	Number of studies included in review (Relevant studies*)	Main findings	Conclusions
Bhangu *et al.* (2023)	International	Systematic review (/-December 31, 2021)	Prioritise feasible interventions to reduce the environmental impact of operating theatres.	Search terms included ‘carbon footprint’, ‘greenhouse gas emissions’, ‘climate change’, and ‘net-zero’, plus ‘surgery’, ‘operating room’ and ‘anaesthesia’	36 (13)	43 interventions were identified, which had low uptake in practice according to 3042 professionals globally	This review identifies actionable interventions applicable to both high-income countries and low-income countries middle-income countries
Bolten *et al.* (2022)^17^	Netherlands	Scoping Review (/-June 2021)	Synthesise evidence for the carbon footprint of commonly used infection prevention measures in the OR, namely medical devices and instruments, surgical attire and air treatment systems	Synonyms for GHG emissions, infection prevention measures and ORs. Studies were included if they described the potential impact of infection prevention measures in the OR on the carbon footprint of the hospital	57 (20)	Many infection prevention measures result in increased emissions. Use of disposable items instead of reusable items increases carbon footprint. Controversy re: correlation between air treatment systems, contamination and incidence of surgical site infections (SSIs)	Use of new air treatment systems and disposable items can lead to higher emissions, and may not reduce rates of SSIs. Alternatives are available. Suggestion to add environmental impact as an additional dimension of quality of care
Bravo *et al.* (2022)^16^	United States	Literature review (Not reported)	Summarise the current, available literature related to the cost efficiency and environmental impacts of wide-awake, local anaesthesia, no tourniquet (WALANT) hand surgery	Directly related to the financial and/or environmental effects of wide-awake hand surgery (WALANT), regardless of the surgical setting, what country the study took place in, or the level of evidence	26 (5)	Found benefits of WALANT to be: (1) Cost savings from local vs general anaesthetic, surgery setting, anaesthesia services, open CTRs vs endoscopic CTRs, shorter recovery room times after local anaesthetic; (2) Time efficiency: decreased surgical time, room turnover time, and time spent in the post anaesthesia care unit; (3) OR waste reduction	WALANT hand surgery more cost effective and environmentally friendly than traditional hand surgery with sedation. By using this technique, surgeons can reduce the national healthcare economic burden while continuing to provide high-level patient care
Drew *et al.* (2021)^22^	Canada	Literature review (/-May 15, 2020)	Summarise state of LCA practice as applied to surgical and anaesthetic care via review of literature assessing environmental impacts of related services, procedures, equipment, and pharmaceuticals	Combined search terms relating to LCA with those relating to surgery and anaesthesiology	44 (22)	Annual climate impact of operating surgical suites ranged between 3 200 000 and 5 200 000 kg CO_2_e. Climate impact of individual surgical procedures varied; estimates ranged from 6 to 1007 kg CO_2_e. Anaesthetic gases, single-use equipment, and HVAC were main emissions hotspots. Single-use equipment used was generally more harmful than equivalent reusable items	Methodological heterogeneity, external validity, and a lack of background life cycle inventory data related to many essential surgical and anaesthetic inputs are key limitations of the current evidence base
Guetter *et al.* (2018)^15^	United States	Narrative review (/-September 2017)	Review the available literature on tactics to reduce OR waste and the potential of these strategies to impact the environment.	Search terms combined with ‘operating rooms’ and ‘surgical theaters’: e.g. ‘green’, ‘waste reduction’, ‘reuse’, ‘recycle’, ‘sustainable’, ‘repurposing’	37 (2)	Summarises literature on reduction, reusing, recycling, rethinking, and renewable energies. Found that one of the most relevant obstacles to executing new plans of action consist in adapting them to the current OR routine, always being attentive to achieving the best patient healthcare	Comprehensive narrative review of current practices and opportunities to improve sustainable OR practices that could reduce waste, contribute to global surgery initiatives, and contain costs.
Kwakye *et al.* (2011)^18^	United States	Literature review and expert interviews (January 1, 1980-December 31, 2008)	Identify leading practices to promote environmentally friendly and efficient efforts in the provision of surgical healthcare	Three main categories: (1) environmental problems, (2) interventions, (3) results	43 (0)	Five strategies were agreed on as the highest-priority solutions for the surgical community. These were: (1) OR waste reduction and segregation, (2) reprocessing of single-use medical devices, (3) environmentally preferable purchasing, (4) energy consumption management, and (5) pharmaceutical waste management	Need for better and more widespread environmentally friendly initiatives. The field of surgery represents a high-yield area for which green practices can be implemented, often with associated cost savings
Lam *et al.* (2023)^40^	UK	Systematic review (/-February 2, 2022)	Systematically evaluate interventions designed to improve the sustainability of surgical practice with respect to their environmental and financial impact.	Search terms included ‘sustainability’, ‘carbon footprint’, ‘environment’, ‘planetary health’, ‘climate change’, ‘surgery’, ‘interventions’, ‘minimise’	21 (17)	Interventions categorised into: ‘reduce and rationalise’, ‘reusable equipment and textiles’, ‘recycling and waste segregation’, ‘anaesthetic alternatives’, and ‘other’. 11 studies examined reusable devices; those demonstrating a benefit reported 40–66% lower emissions than with single-use alternatives. In studies not showing a lower carbon footprint, the reduction in manufacturing emissions was offset by the high environmental impact of local fossil fuel-based energy required for sterilisation. Per use monetary cost of reusable equipment was 47–83% of the single-use equivalent	A narrow repertoire of interventions to improve the environmental sustainability of surgery has been trialled. The majority focuses on reusable equipment. Emissions and cost data are limited, with longitudinal impacts rarely investigated. Real-world appraisals will facilitate implementation, as will an understanding of how sustainability impacts surgical decision-making
Mubarak *et al.* (2023)^19^	UK	Systematic review (May 2017 - June 2022)	Critically evaluate sustainable healthcare to provide quality surgical care in the United Kingdom.	Relevant articles addressed the sustainability and performance of the healthcare system, including sustainability drivers; frameworks or policy responses for improved sustainability; and formulation, implementation, and evaluation of relevant interventions	15 (3)	Ten articles evaluated existing sustainability practices. Seven articles discussed significant determinants of quality healthcare. Nine articles highlighted implications of sustainability. Conserving water, optimising treatment routes and transportation, and creating cultural change were found to be the pillars of high-quality, sustainable healthcare	The concept of sustainability varied between these studies. Anaesthetic gas emissions from ORs continue to have the most detrimental effect on the sustainability of the surgical industry. A significant gap was noted between the available data and their implications
Perry *et al.* (2022)	UK	Systematic review (/-March 2, 2020)	Assimilate the published studies concerning the sustainability of the perioperative environment, focussing on the impact of implemented interventions.	Clearly describe the interventions or processes to improve the sustainability of operating theatres	34 (34)	Demonstrates interventions which could be implemented to improve sustainability within the perioperative environment. OR personnel are crucial to this. Within anaesthetics, the use of smaller propofol vials reduced waste, the theatre staff turning off idle machines reduced electricity use, the surgeons and scrub staff reviewing and streamlining the theatre packs reduced waste	There are successful and straightforward interventions that can be implemented that do not compromise patient safety. The initiatives highlighted in this review are easy to implement and could reduce significant volumes of emissions by avoiding wasteful practices
Pradere *et al.* (2022)	France	Systematic review (January 1, 1990-April 1, 2021)	Review of the available actions that could limit CO2 emissions in the operating room (OR) and their potential benefits upon the environment, whilst preserving quality of care	Actions that take place in the OR that assessed any climate-smart actions that aimed to improve the sustainability of surgical activities. Collected all variables that objectively reflect climate-smart actions, e.g. CO_2_e, GHGe	38 (16)	Identified six core climate-smart actions: (1) waste reduction by segregation; (2) waste reduction by recycling, reuse, and reprocessing; (3) sterilisation; (4) anaesthesia gas management; and (5) improvement of energy use. Waste management is the area where healthcare workers could have the strongest impact, whereas the main field to reduce CF in the OR was found to be energy consumption	Educational programmes should be implemented. Reducing waste production, improving segregation, and recycling protocols are the easiest actions to implement. Further multidisciplinary consensus is needed to provide quality endpoints to use in this setting, and to improve reporting
Reynier *et al.* (2021)^38^	France	Narrative review (January 2000-April 2020)	Does use of disposable or reusable medical devices lead to a difference in terms of: hospital acquired infection or bacterial contamination, financial cost, and environmental impact (carbon footprint, energy, water, waste)	Search terms, e.g. ‘reusable’, ‘disposable’, ‘infection’, ‘operating room’, ‘life cycle’ plus medical devices commonly used in the anaesthesia work area e.g. ‘scrubs’, ‘masks’, ‘gloves’, ‘laryngoscope blade and handles’, ‘blood pressure cuff'	81 (7)	According to this review, the following improvements could be implemented in the OR to improve sustainability of our work as anaesthetists: use reusable cloth skull caps, scrubs, sterile gowns, monitoring devices, laryngoscopes blades and handles; improve the disinfection of the work area between patients; improve hand hygiene in the anaesthesia work area	Disposable medical devices and attire in the operating theatre do not mitigate the infectious risk to the patients but have a greater environmental, financial and social impact than the reusable ones
Rizan *et al.* (2020)^7^	UK	Systematic review (/-October 4, 2019)	Evaluate existing literature calculating the carbon footprint of surgical operations, determining opportunities for improving the environmental impact of surgery	Studies evaluating the carbon footprint of individual surgical operations	8 (8)	This review found that the carbon footprint of a single operation ranged from 6 (for cataract surgery in India) to 814 kg CO_2_e, (for a robotic hysterectomy in the US), with the largest value being equivalent to driving up to 2273 miles in an average petrol car. This review found that the major carbon hotspots within operating theatres are: energy use and procurement of consumables (especially single use items)	Optimum approach to reducing emissions of a given operation is holistic, including electricity use, anaesthetic gases, and use of equipment, especially where disposable. Also, consider reducing need for surgery through health promotion, disease prevention, and correct patient selection
Shoham *et al.* (2022)^44^	Canada	Systematic review (/-June 23, 2020)	Assess and summarise the published evidence of the environmental impact of surgery	Studies that systematically or quantifiably reported environmental impact for a particular surgery or surgical specialty was included	55 (26)	Using anaesthetic gases with low global warming potential reduces OR emissions without compromising patient safety. OR waste is often disposed of improperly, often due to convenience or knowledge gaps. There are environmental benefits to replacing disposable materials with reusable equivalents, and to proper recycling. Surgeons can help implement these changes at their institution	Although there is a clear need to lower the carbon footprint of surgery, the quality of research with which to inform protocol changes is deficient overall. This attempt to quantify surgery’s carbon footprint yielded heterogeneous data and few standardized, actionable recommendations
Shum *et al.* (2022)^42^	Australia	Systematic review (/-July 2022)	Review the current knowledge of Interventional Radiology (IR) waste generation and ways of reducing waste in practice, to quantify the environmental and financial impact of waste generated and address green initiatives to improve IR waste management	Articles focused on waste generation and management in IR practice were targeted, keywords used include ‘radiology’, ‘interventional radiology’, ‘endovascular’, ‘recycling’, ‘waste’, ‘environment friendly’, ‘sustainability’, ‘greening’, ‘cost’, ‘climate change’, ‘global warming’, ‘operating theatre’, and ‘operating room’	68 (14)	Interventional radiologists can engage with suppliers to reformulate procedure packs to minimise unnecessary items and packaging. Opened but unused equipment can be prevented if there is better communication within the team and increased staff awareness of wasted equipment cost. Incentives to use soon-to-expire equipment can be offered. Power consumption can be reduced by powering down OR lights and workstations when not in use, changing to Light Emitting Diode (LED) and motion sensor lights. Hand wash can be replaced with alcohol-based hand rubs to reduce water usage. Barriers to improving waste management include lack of leadership, misconceptions regarding infectious risk, lack of data, concerns about increased workload, negative staff attitudes and resistance to change	Interventional radiologists have a crucial role to play in improving healthcare sustainability. By implementing small, iterative changes to our practice, financial savings, greater efficiency and improved environmental sustainability can be achieved. Education remains a top priority to engage all staff in sustainable healthcare practices
Sullivan *et al.* (2023)^39^	United States	Scoping Review (/-February 2022)	Identify quality improvement initiatives that aimed to reduce the environmental impact of the OR while decreasing costs	Included broad terms for the ‘operating room’, ‘costs’, and ‘environment’ or ‘sustainability’ was used	23 (13)	A total of 23 unique quality improvement initiatives describing 28 interventions were included. Interventions were categorised as ‘refuse’, ‘reduce’, ‘reuse’, and ‘recycle’. Methods of measuring environmental impact and cost savings varied greatly. Potential annual cost savings ranged from $873 to $694 141	Quality improvement initiatives that reduce both cost and environmental impact have been successfully implemented
Wyussek *et al.* (2019)	Australia	Narrative review (/-2018)	Focus on the trend of OR greening initiatives over the last 25 y, comparing different innovative approaches, the successes and setbacks, and the financial implications of initiatives	Identify articles which focused on hospital and operating theatre waste management using keywords e.g. ‘operating rooms’, ‘waste management’, ‘environmental impact’, ‘review’	Not reported (9)	Measures that healthcare personnel can take to reduce the ecological footprint of their healthcare facility outlined, e.g. reducing, recycling, reusing, rethinking and researching, novel technology and smarter architectural design. Evaluated barriers to improving waste management, e.g. lack of leadership, misconceptions among staff, and an overall resistance to change	Hospitals must reduce the environmental impact of their facility

‘Relevant studies’ included in this column were determined by the authors to have the following: an environmental focus, intervention/ comparison, not reviews or reports, are published, not conference abstracts, focused on surgery/operating theatres, must be comparing at least two options if a comparison study.

**Figure 2:**
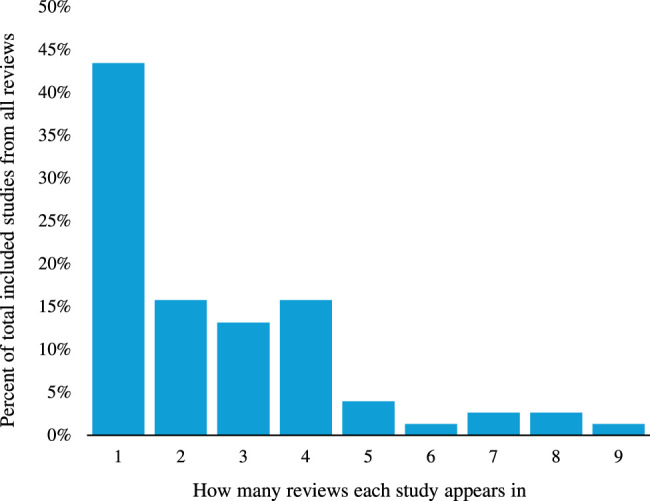
Percentage of included studies appearing in multiple reviews.

A total of 76 studies were included in these 16 reviews (43% of these appeared in one review as illustrated in Fig. 2). Nine (12%) studies appeared in five or more reviews. These studies were separated into tested interventions, and predicted interventions (e.g. LCAs).

### Categorising the interventions according to the five Rs and the BCW

Of the 73 individual studies covered by the reviews, 30 tested interventions. The outcome data did not allow meaningful comparisons between interventions as they were measured using different metrics and relied on local factors, such as service volume and supply chains, making comparisons difficult and potentially misleading. Since studies used different metrics, percentages, and measured their effects over different, often unreported, periods of time. Consequently, the aim to compare impact of interventions across studies was abandoned. Instead, interventions were either considered ‘effective’ if they had a positive outcome or ‘ineffective’ if they had a negative outcome or failed to make a change in staff behaviour.

Of the 30 studies, only two^46,47^ were ineffective (one in reduce and one in reuse categories). Fourteen of the 30 studies aimed to reduce the use of resources, 6 aimed to initiate or improve recycling, 6 aimed to change practice (refuse), 3 reusing items, 1 renew through repurposing and 3 aimed to achieve a combination (multiple sustainability targets). The interventions and their various outcomes are detailed in Table 3.

**Table 3:** Interventions divided by circular economy concept and categorised according to the behaviour change wheel (BCW).

Main concept(s)	Study	BCW		Intervention category	Outcomes			
		Intervention functions	Policy category		Waste	Emissions or CO_2_e	Financial	Other
Refuse	Barwise *et al*.^65^	Environmental restructuring	Guidelines	Upgrading technology infrastructure				The new system produces energy savings and may increase vacuum pump lifespan.
	DeBois *et al*.^66^	Environmental restructuring	Guidelines	Changing local protocols	Almost 15 000 pounds (7.5 tons) of trash will be diverted from RMW	This process not only releases a significantly less amount of carbon dioxide into the environment, but also helps generate renewable energy		
	Van Demark *et al*.^67^	Environmental restructuring	Guidelines	Changing local protocols	Surgical waste was decreased by 5.06 pounds per case		Cost savings for the new ‘green packs’ was $10.64 per case. The overall cost savings was $13 250.42	
Reduce	Park *et al*.^48^	Education	Communication/ marketing	Education on financial and environmental cost	reduced the use of disposable trocars by 56% and the use of disposable harmonics and staplers by 33%		Reduced median supply cost per case by 43%, with total cost savings of $71 035 for the first four quarters	
	Southorn *et al*.^50^	Education	Communication/ marketing	Education on waste segregation	Clinical waste reduced by up to 5.8 kg an operation	75% lower carbon footprint		
	Mosquera *et al*.^60^	Training	Communication/ marketing	Training on waste segregation	Significant reduction in the monthly average healthcare waste volume of 6.2%		Savings cost of €125 205	
	McCarthy *et al.* ^46^	Education	Communication/ marketing	Education on financial and environmental cost				No improvement was found 18 mo later
	Perrego *et al*.^49^	Education	Communication/ marketing	Education on waste segregation	41% reduction in the total mass of regulated waste sampled and a 77% reduction in nonregulated item mass			
	Mankes *et al*.^59^	Restriction	Guidelines	Restriction of supplies	Eliminating the 50/100 ml bottles reduced propofol waste from 29.2 ml/d/bin to 2.8 ml/d/bin			
	Hubbard *et al.* ^52^	Environmental restructuring	Guidelines	Changing policy and physical infrastructure	Annual reduction in medical waste of ~8470 kg		Cost savings of $2200 per year	
	Zuegge *et al.* ^58^	‘Education Environmental restructuring’	Communication/ marketing	Intervention package		CO_2_ equivalent emissions per case dropped from163 kg to 58 kg (64% reduction)		55% reduction in desflurane usage
	Martin *et al*.^57^	‘Education Environmental restructuring’	Communication/ marketing	Intervention package	‘Daily landfill waste reduced by 12%. Weight of medical waste reduced by 59% Recyclable waste increased by 19%’			
	Denny *et al.* ^55^	‘Education Environmental restructuring’	‘Guidelines Communication/ marketing’	Intervention package	63% decrease of weekly waste for endotracheal tubes, a 54.7% reduction in laryngoscope handles waste, and 54.0% reduction laryngoscope blades			
	Fraifeld *et al.* ^56^	‘Education Environmental restructuring’	Communication/ marketing	Intervention package	Decrease in overall weight of regulated medical waste items from 0.33 kg/case to 0.09 kg/case (*P*<0.001); segregation audit showed overall increase in correctly segregated regulated waste of 65%		Cost savings of $15.60 per OR per week, or $28 392 annually	
	Thiel *et al*.	Environmental restructuring	Guidelines	Changing policy and physical infrastructure	13% less waste produced.		55% cost reduction	
	Lin *et al.* ^53^	Environmental restructuring	Environmental/ social planning	Upgrading technology infrastructure				50% of energy saving in the OR with this system
	French *et al*.^51^	‘Education Environmental restructuring Enablement’	‘Guidelines Communication/ marketing’	Intervention package	In 2 year - 30 000 lbs of waste reused/recycled			
Reuse	French *et al*.^51^	‘Education Environmental restructuring Enablement’	‘Guidelines Communication/ marketing’	Intervention package	In 2 year - 30 000 lbs of waste reused/recycled			
	Proctor and Raym^47^	Education	Service provision	Education through sustainability committee	Decided to keep single-use gowns, but did institute a disposable wrap recycling programme			
	Boone *et al.* ^68^	‘Education Environmental restructuring Enablement’	‘Service provision Guidelines’	Intervention package	5 year later, saved more than 9000 lbs of waste		5 year later, more than $251 000 in cost savings resulted from the reprocessing programme	Now part of the culture of the surgical team and a ‘nonissue’
Recycle	Wyssusek *et al*.^43^	Environmental restructuring	Guidelines	Intervention package	reduced the amount of clinical waste produced by the OR by 82%, and the amount of total OR waste was reduced by more than 50%		60% reduction in costs	
	French *et al.* ^51^	‘Education Environmental restructuring Enablement’	‘Guidelines Communication/ marketing’	Intervention package	In 2 years - 30 000 lbs of waste reused/recycled			
	Babu *et al*.^61^	Environmental restructuring	Guidelines	Changing local protocols	1247 lbs waste collected. 31.2 cubic feet landfill saved		Cost avoidance yielded $31 680.00 in savings	376.75 gallons of oil saved. 3175.7 kWH electricity saved
	Francis *et al*.^63^	Environmental restructuring	Guidelines	Changing policy and physical infrastructure	30% recovery of non- infectious waste			
	McKendrick *et al*.^64^	Environmental restructuring	Guidelines	Changing policy and physical infrastructure	‘Across 20 operations 54 kg of recyclable waste (50% of AR and 67% of OR waste)’	The 54 kg of recycled bags produced during the study saved 25 kg CO_2_ emissions	Recycling saved a mean of £0.51 per case.	
	Bliss *et al*.^62^	Environmental restructuring	Guidelines	Changing policy and physical infrastructure	Infectious waste reduced by 3.4 kg per procedure		Contaminated or infectious waste costs $5.60 per red bag and noninfectious waste costs $0.02 per pound, decreasing both forms of waste is economically attractive to hospitals	
Renew	Tay *et al*.^69^	Environmental restructuring	Environmental/ social planning	Upgrading technology infrastructure		Greenhouse gas emissions decreased by 44%	volatile agent cost was $18.87/hour using manual control and $13.82/hour using automated control: mean decrease $5.05/hour	The 100-year global warming potential decreased from 23.2 kg/h of carbon dioxide equivalents to 13.0 kg/h: mean decrease 10.2 kg/h
Multiple sustainability targets	Wormer *et al.* ^71^	‘Education Environmental restructuring Enablement’	‘Service provision Guidelines Environmental/social planning’	Intervention package	Recycling reduced waste by 12 860 lbs per year. New bins = 5443 kg per year recycled, 75% reduction in red-bag waste	Turning off equipment reduced Co_2_ emissions by 234.3 metric tons per year	Switching foam padding saved $50 000 per year. Turning off equipment saved $33 000 per year	Scrubbing with gel instead of washing hands saved 2.7 million litres of water per year
	Burrell *et al.* ^70^	‘Education Enablement Restriction’	‘Service provision Communication/marketing Guidelines’	Intervention package		A third less greenhouse gas emissions		Decrease in the volumes of desflurane used
	Albert *et al*.	Environmental restructuring	Guidelines	Intervention package	Recycling increased from 0% to 20–50%. Repurposing of 1.2 tonnes of blue wrap			

**Figure 3:**
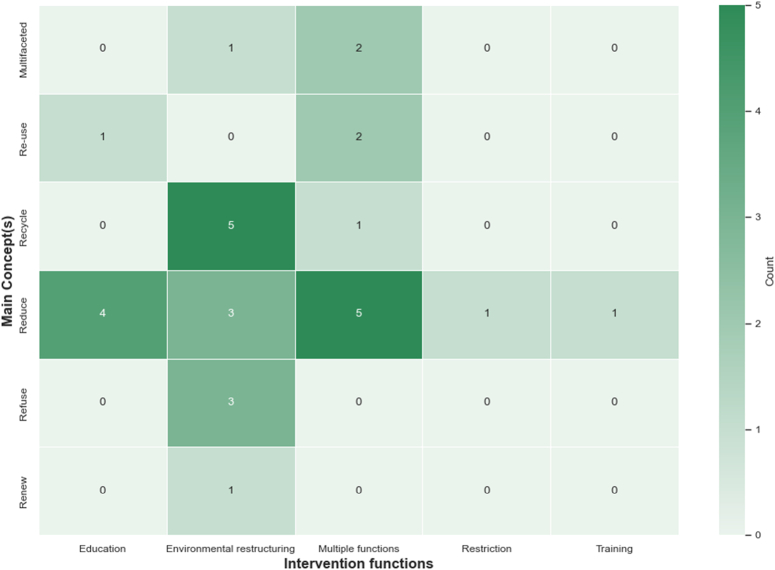
Matrix chart mapping sustainability concepts and IFs. Most studies aimed to ‘reduce’ (*n*=14) and ‘recycle’ (*n*=6). Multiple IFs (education + environmental restructuring without (*n*=4) or with (*n*=1) enablement) were successful in reducing surgical waste (*n*=5) followed by ‘education’ and ‘environmental restructuring’ which were successful single interventions (4 and 3, respectively), while ‘environmental restructuring’ was the single most intervention employed to increase recycling in surgery (*n*=5).

As illustrated in Table 2, these studies were mapped to five of the nine IFs and four of the PCs described in the BCW. ‘Environmental restructuring’ and ‘education’ were the most utilised (16 and 5 studies, respectively), followed by a combination of IFs (utilised in 10 studies). These mostly were: ‘education’ and ‘environmental restructuring’ (*n*=4), ‘education’, ‘environmental restructuring, and ‘enablement’ (*n*=5), and ‘education’ and ‘environmental restructuring’, and ‘restriction’ (*n*=1). ‘Restriction’ and ‘training’ IFs were each reported in a single study. Figure 3 is a heatmap that illustrates the interplay between various IFs and the five Rs.

#### Reduce

Reducing surgical waste was the most common strategy identified in this review, and was successful in 13 of 14 studies (92.8%). The predominant approach involved incorporating both ‘education’ and ‘environmental structuring’ as multiple IFs which was successful in five (100%) studies, ‘education’ as the sole IF was successful in three out of four (75%) studies, ‘environmental restructuring’ as the sole IF in three (100%) studies, ‘restriction’ in one and ‘training’ in another (each 100% effectiveness).

### Education

Four studies^46,48–51^ employed interventions that relied on education (3; 75% were successful). Interventions included educating surgeons on the costs of supplies used in a procedure^48^, waste segregation^49,50^, and on the financial and environmental benefits of turning off computers at night and/or weekends^46^. The latter^46^ was delivered through a one-off session and was effective at first, but the impact was not retained 18 months later.

### Environmental restructuring

Three studies^52–54^ focused on changing the physical environment and context within OTs to achieve their aims (all of them were successful). Interventions included introducing noncontaminated waste bins in anaesthesia rooms, making bins accessible, and regulating their use to reduce the amount of landfill waste^52^. Other strategies included installing motion sensors to control ventilation systems thereby reducing energy waste^53^, and streamlining packs^54^. It is notable that environmental sustainability outcome data were not reported in relation to streamlining packs, underscoring the complexities of assessing the environmental impact of this intervention.

### Multiple IFs (education and environmental restructuring)

Five studies^51,55–58^ reported the use of intervention packages centred around education and environmental restructuring (all of which were successful). One intervention included staff education (with a focus on new employees) and labelling items with their environmental impact^58^. In addition to staff education, interventions focused on optimising and signposting at anaesthesia workstations, introducing pharmaceutical waste containers in each OT^56^, changing waste bin designs, and using labels and signage for better segregation^57^. Finally, Denny *et al*.^55^ aimed to reduce the waste and increase cost savings of opened and unused endotracheal (ET) tubes and disposable laryngoscope handles and blades. The intervention package included education (visual presentations of the collected opened and unused ET tubes and laryngoscope handles and blades), new practice guidelines, and flyers containing the same photos and guidelines in key locations.

### Restriction

Mankes^59^ successfully reduced pharmaceutical waste in operating rooms by restricting access to 50 and 100 ml vials of propofol from the pharmacy inventory, and retaining only the smallest size (20 ml), which reduced propofol waste by 90%.

### Training

Mosquera *et al*.^60^ focused on waste reduction through training sessions for proper waste sorting and disposal, thereby reducing the monthly average healthcare waste volume by 6.2%.

#### Recycle

All six studies^43,51,61–64^ in this category were effective in improving recycling in surgery. ‘Environmental restructuring’ was employed to: (i) improve segregation by introducing a recycling programme for the general waste^43^, (ii) recycle blue wrap externally^61^, (iii) introduce facilities to allow recycling of uncontaminated packaging materials^62^, (iv) address incorrect waste labelling (labelling waste as infectious or contained material)^63^, and (v) install dedicated paper and cardboard recycling bins^64^. French^51^ relied on multiple IFs (education, environmental restructuring, and enablement) to reduce the amount of waste through changing bin colours, informative labelling, staff education, and regular online reminders for staff in line with the US Environmental Protection Agency’s guidelines. The project identified and addressed the staff’s perception and concerns about infection control by correcting misconceptions through education. Phase 3 of the project focused on recycling through segregation, and education (especially for new employees and medical students).

#### Refuse (rethink)

All three interventions^65–67^ were effective in changing standard practices to adopt more sustainable alternatives using ‘environmental structuring’. This was done by diverting cardiopulmonary bypass circuits’ waste from the traditional regulated medical waste to clear bag waste, or municipal solid waste through changing local processes, thereby reducing the impact of waste processing^66^. Other interventions included performing certain operations under regional anaesthesia instead of general anaesthesia, thereby reducing the use of resources^67^ or using low-flow scavenger interface machines^65^.

#### Reuse

Three studies^47,51,68^ fell in this category; two were effective (66%). The effective interventions employed multiple IFs (education, environmental restructuring, and enablement) as a package^51,68^. Boone and Penny^68^ created a sustainability committee that introduced the use of reprocessed surgical devices, educated and addressed users’ concerns about safety, quality, and reliability. Similarly, the second phase of French^51^ changed local policy to allow the reuse of items which had been opened, but not used in a surgical field for reuse in other departments that do not require OT-level sterility. The ineffective intervention attempted ‘education’ as the sole IF^47^. Proctor and Raym^47^ educated the staff on the use of reusable gowns with the help of a sustainability committee; however, despite revealing potential annual savings of $10 000, the shift to single-use gowns did not materialise.

#### Renew

One study^69^ fell in this category and was effective in reducing cost by 27% and GHG emissions by 44% by replacing old anaesthesia machine with newer ones that have automated control of gases’ functionality.

### Multiple sustainability targets

Three studies^70–72^ implemented intervention packages to target more than one element of the five Rs using multifaceted-approach interventions (multiple IFs); all of them were effective. Two interventions used education, environmental restructuring, and enablement. Wormer *et al*.^71^ aimed for the reduce and reuse strategies simultaneously and successfully employed a sustainability committee that facilitated replacing single-use items with reusable ones, reducing energy use, and switching to waterless scrub. Similarly, Albert and Rothkopf^72^ employed a committee to enable the streamlining of surgical trays, the initiation of a paper/plastic recycling programme, and the establishment of a blue wrap repurposing scheme through environmental restructuring. However, it is noteworthy that while the study saved an estimated $41 844 per year, the environmental impact of streamlining trays was not reported. Finally, Burrell^70^ aimed to reduce and refuse through ‘education, environmental restructuring, and persuasion’. The intervention involved extensive repeated education, designating sevoflurane as the default anaesthetic gas, and reports of monthly calculations of the carbon footprint. Table 3 illustrates the identified interventions according to the five Rs, and categorised according to the BCW’s IFs and PCs.

## Comparison studies

The remaining 43 studies included in the reviews were comparison studies, which estimated and predicted the outcome of two alternatives (such as reusable compared to disposable versions of the same equipment). The findings were largely based on LCAs illustrating a potential effect rather than an actual one. Analysing these comparison studies based on their country of origin is crucial due to the differences in supply chains, healthcare practices, waste management systems, and environmental regulations across nations. MacNeil *et al*.^73^ showcased the differences in practices between healthcare systems, which underscores the caveats associated with LCA studies. The study calculated the estimated carbon footprints of surgical operations across 1 year in 3 comparable surgical suites in the UK, Canada, and US^73^. Their results showed that there is a 10-fold difference in anaesthetic gas emissions between the three comparable sites. This difference resulted in the carbon footprint of an operation in Canada to be 18% lower than the same operation in the UK, and 56% lower than the USA. Consequently, central strategies were discussed in their specific context, with emphasis on transferable practices. Table 4 illustrates the full list of strategies and their relevant impact for reference.

**Table 4:** Comparisons categorised by country or region.

Country	Study author and year	What was compared or studied	Outcomes		
			Emissions or CO_2_e	Financial	Other
Australia	McGain 2012^76^	Reusable vs disposable: Central venous catheter insertion kits	CO_2_ emissions = 1211 g reusable, 407 g single use	Reusable cost $6.35 Australian; disposable cost $A8.65	Water consumption = 27.7 l reusable, 2.5 l single use
	McGain 2010	Reusable vs disposable: Anaesthetic drug trays	CO_2_ production = 110 g reusable, 126 g single use (15% more)		Water usage = 3.1 l reusable, 10.4 l single use
	McGain 2016^80^	Sterilisation: Different steam sterilisers	Sterilisers were idle 48% of the time. Switching off when idle would save ~79 tonnes of CO_2_ emissions a year		Switching off when idle would decrease electricity by 26% and water by 13%
	McGain 2017b	Sterilisation: Active vs. idle steam sterilisation unit			40% of electricity (60% when active) and 21% of water use (79% when active) were whilst the machine was idle. Heavier loads were more efficient
	McGain 2021^78^	Other: Use of spinal anaesthesia with sedation (SA) / combined general anaesthetic and spinal vs. Use of general anaesthetic (GA) (either volatile or total intravenous, i.v.)	Emissions similar for GA, SA, and combined approaches (14.9 vs. 16.9 vs. 18.5 kg CO_2_ eq). Electricity for air warmer = 20% for GA, 21% SA, and 19% combination. Sevoflurane = GA (35%) and combined (19%) emissions. Washing and sterilising reusable items = SA (29%), combined (24%), and GA (4%). Oxygen was key to the SA carbon footprint (18%)		
	Davis 2018^75^	Reusable vs disposable: Flexible uretroscope	Carbon footprint of LithoVue = 4.43 kg CO_2_ per endourologic case. Carbon footprint of reusable ureteroscope = 4.47 kg of CO_2_ per case		
Australia, UK/Europe, USA comparison	McGain 2017a	Reusable vs. disposable: Anaesthetic equipment	Conversion of single-use to reusable equipment: Australian hospitals = increased emissions 5095 kg CO_2_eq +9%, UK hospitals = reduction of 4293 kg CO_2_ eq, 84%, USA = reduction of 2427 kg CO_2_ eq, 48%	Annual financial cost of converting from single-use equipment to reusable anaesthetic equipment would be an AUD$32 033 (£19 220), 46% decrease	
Canada	Lui 2014	Other: Recycling differences in different head and neck surgery subspecialties			Waste - paediatric produced the least recyclable material per operation as a proportion of total waste, which was statistically different than the two highest recyclable subspecialties, general and rhinology. Recycling these recyclables could eliminate 21% of operating room waste mass
Canada, USA, UK	MacNeill 2017^73^	Other: Countries of Canada vs. USA vs. UK	VGH (Canada) = 146 kg CO_2_e per operation; JRH (UK) = 173 kg CO_2_e; UMMC (USA) = 232 kg CO_2_e. Overall carbon footprint of surgery in the three countries = 9.7 million tonnes CO_2_e per year		Preferential use of desflurane = 10x difference in anaesthetic gas emissions between hospitals. OT 3-6x more energy-intense than hospital as a whole (due to heating, ventilation, air conditioning)
Chile	Berner 2017	Surgeries: Abdominoplasty vs. bilateral breast augmentation vs. rhinoplasty	Rhinoplasty = total carbon footprint of 16.99 kgCO_2_e; Breast = 16.23 kgCO_2_e; Abdominoplasty = 23.68 kgCO_2_e		
Germany	Leiden 2020^84^	Reusable vs. disposable: Instrument sets for lumbar fusion surgery	Cleaning and sterilisation process for reusable instruments is responsible for up to 90% of GHGe		For all impact categories the reusable system has a higher negative environmental impact
	Adler 2004	Reusable vs. disposable: Instruments for laparoscopic cholecystectomy		Performance of laparoscopic cholecystectomy with disposable instruments = 19x more expensive than for reusable instruments	Reusable instruments are environmentally advantageous
	Kummerer 1996	Reusable vs. disposable: Laparotomy pads (swabs)	Disposable laparotomy pads have higher emission of nitrogen oxides (1637.0 g v. 102.9 g)		Disposable laparotomy pads have higher energy consumption (6704.8 MJ v. 2431.8 MJ)
Germany/Australia	Ibbotson 2013^82^	Reusable vs. disposable: Stainless steel, fibre-reinforced plastic, reusable stainless steel scissors		Total cost of ownership over 4500 cycles: disposable (plastic) = €14 085; disposable (stainless steel) = €12 375; Reusable = €7811	Environmental impacts of the disposable steel = 80% worse than disposable plastic, 99% worse than reusable steel scissors
Netherlands	Traversari 2017^86^	Ventilation: Switching off during prolonged inactivity (during the night and weekend)		Saves money	No negative effect on air quality in OTs during normal operational hours. Can save 70% of energy consumption vs systems on full capacity continuously
Spain	Tejero-Gonzalez 2020	Ventilation: Setback vs. normal			Heating + electric yearly supply energy savings = 29 MWht and 262 Mwhe (2% of the total electric energy consumption of the hospital) when using setback
UK	Lee 2018^92^	Ventilation: Different rates		70% of reduction in annual energy cost when reducing ventilation rate from 30 to 6 ACH	
	Gatenby 2011^87^	Surgeries: Gastro-oesophageal reflux disease: surgical vs. medical management	100 Kg CO_2_ per year in the medical arm; 30 Kg CO_2_ per year in the surgical arm	Surgery cost = £2039; Medical treatment = £309	
	Jehle 2008^91^	Scrubbing: Scrubbing vs. alcohol-based preparation			18.5 l of water used per staff member scrubbing. Could be saved with alcohol preparations
	Weiss 2016^95^	Scrubbing: ‘Knee on’ vs. ‘elbow on’ taps for surgical scrubbing			‘Elbow on’ taps lead to a mean increase of “water on” time whilst scrubbing by 1 min16 s. Equating to 5.7 l of hot water
	Rizan 2022^93^	Sterilisation: Comparing sterilisation of different instrument sets	‘Decontamination + packaging instruments = 66–77 g CO_2_e per instrument in the set. Individually wrapped instruments = 189 g CO_2_e per item. Removing items from a set proportionally increased footprint, with an increase on average of 38 g CO_2_e per item removed across all operations requiring the streamlined set’	Cost of decontamination + packaging = €1.05–€1.07 per instrument in containers; €7.35 per individually wrapped instrument	
	Jabouri & Abbott 2021	Reusable vs. disposable: Instrument sets for skin surgery	‘Emissions greater for single-use vs. reusable sets (1.436 vs. 1.121 kg CO_2_e). Sterilisation (40.6%), production (37.2%) + disposal (22.2%) contributed to emissions for reusable packs. Production (62.6%) + disposal (37.4%) contributed to single-use set emissions’	Cost of single-use sets = £20.57 vs £13.35 for reusable devices, per use based on a weekly usage. Based on average weekly use = equates to £50 659.54 per year	
	Rizan & Bhutta 2022^93^	Reusable vs. disposable: Tray wraps/flexible pouches, rigid aluminium containers	Carbon footprint per instrument for single-use tray wrap containers was lower than reusable aluminium or flexible pouches (13 vs. 25 vs. 44 g CO_2_)	Using a combination of hybrid laparoscopic instruments cost less than half of single-use equivalents (GBP £131 vs £282)	
	Moussa 2022^88^	Surgeries: Air tamponade (AT) vs. Fluorinated gases in repairing selected rhegmatogenous retinal detachment (RRD)	Fluorinated gas systems = 63x higher CO_2_ emissions per repair vs. air tamponade. The hospital which used air tamponade in 70% of RRD repairs had 47.0% and 41.1% lower emissions vs. hospitals using fluorinated gas. Assuming 30% of repairs are suitable with AT in the U.K., its use could reduce 716.5 tons of CO_2_ annually (a 44.3–56.6% reduction in emissions from RRD repairs)		
	Hu 2021^89^	Other: Anaesthesia - IAGs (sevoflurane, isoflurane, desflurane) vs. intravenous propofol	Carbon footprint of IAGs is minimised when using oxygen/air mix as the carrier gas at the lowest flow rate while applying a vapour capture technology (VCT) - then footprint is similar to propofol. Also, carbon footprint of sevoflurane is not smaller than desflurane if sevoflurane is synthesised from tetrafluoroethylene		
USA	Donahue 2020^101^	Reusable vs. disposable: Acrylic vaginal specula, reusable stainless steel models	Reusable stainless steel grade 304 speculum had a lesser carbon footprint than either the reusable stainless steel grade 316 or the disposable acrylic specula		
	Albert and Rothkopf 2015^72^	Surgery packs: Disposables in plastic and hand surgeries		$17 381.05 could be saved per year with leaner packs. Single-stream recycling saved $3487 per month at the three campuses. Extrapolated savings of $41 844 per year	15 items removed from disposable plastic packs; 7 items removed from hand packs
	Cantlon and Yang 2017^97^	Surgeries: WALANT surgery vs. full operation			Cleaning and turnover times drop significantly. Less time for patient in recovery. Waste generated from a WALANT procedure is a fraction of that generated from the same case in the main OT
	Carr 2019^98^	Surgeries: Open CTR in the OT vs. OSC vs. clinic		Nearly 7x more expensive in OT vs. clinic: Average direct costs = $213.75 for OR, $102.79 for OSC-MAC, $55.66 for the OSC-local, $31.71 for clinic	Average weight of surgical waste was 4.78 kg in the OT, 2.78 kg in the OSC-MAC, 2.6 kg in the OSC-local, and 0.65 kg in the clinic group
	Thiel 2019^54^	Surgery packs: Minimal surgical supplies pack for hand surgery vs. standard pack		Implementing green hand packs concomitantly with WALANT surgery halved surgical material costs	Reduced surgical waste by 13% in wide-awake hand surgery in the ambulatory surgical setting, with use of local anaesthesia and custom minimised hand packs, compared with sedation, local anaesthesia, and standard hand packs in the hospital setting
	Woods 2015^110^	Surgeries: Laparotomy (LAP) vs. conventional laparoscopy (LSC) vs. robotically-assisted laparoscopy (RA-LSC)	The carbon footprint of a RA-LSC ¼ 40.3 kg CO2e/ case ¼ 38% increase over LSC (29.2 kg CO2e/case) and a 77% increase over LAP (22.7 kg CO2e/case)		
	Campion 2012^96^	Surgeries: childbirth as caesarean section vs. natural delivery			For all births, the processes contributing the most to environmental impacts were energy consumption due to HVAC, the end of life impacts of the disposable custom packs, and the production of the disposable custom packs
	Sherman 2018^104^	Reusable vs. disposable: Laryngoscopes	Sterilisation = 400% more GHGe. Single use handles = 25x more GHGe. Single use blades = 6-8x more GHGe		
	Eckelman 2012^111^	Reusable vs. disposable: Laryngeal mask airways	‘Reusable LMAs = 7.4 kg CO2e Disposable LMAs = 11.3 kg CO2e Per 40 uses’		Disposable = more carcinogenic
	Vozzola 2018^108^	Reusable vs. disposable: Isolation gowns	Reusable gowns = 30% reduction in GHGe vs. disposable		Reusable gowns = 28% reduction in energy consumption, 41% reduction in blue water consumption, 93% reduction in solid waste generation vs disposable
	Vozzola 2020^109^	Reusable vs. disposable: Surgical gowns	Reusable gowns = 66% reduction in GHGe vs. disposable		Reusable gowns = 64% reduction in energy consumption, 83% reduction in blue water consumption, 84% reduction in solid waste generation vs. disposable
	Unger 2017^107^	Other materials: Single use medical products containing plastic vs. biopolymers			The significant agricultural inputs associated with manufacturing biopolymers exacerbate environmental impacts.
	Thiel 2015^105^	Robotic and laparoscopic vs. abdominal and vaginal hysterectomy	Robotic (and laparoscopic) hysterectomies had a significantly higher GHG footprint compared to abdominal or vaginal hysterectomies, after removing variance from anaesthetic gas choice		
	McPherson 2019^103^	Other materials: Different container systems	Reduction in GHG by 162.4 MTCO_2_eq (−65.3%) annually		Eliminated 50.2 tonnes of plastic DSC and 8.1 tonnes of cardboard from the sharps waste stream. Of the plastic eliminated, 31.8 tonnes were diverted from landfill and 18.4 from incineration
	Conrardy 2010^99^	Reusable vs. disposable: Drapes and gowns			Reusable gowns = 59–70% reduction in waste (~5 lb per procedure)
	Thiel 2018^106^	Other: Combining multiple interventions for anaesthesia	Minimising material use and using reusable surgical instruments results in GHGe reductions of 50%. Replacement of desflurane reduces GHGs by >25% per laparoscopic case. Switching to propofol reduces GHGs by 28%		Occupancy sensors in the OR reduced the electricity use by a third over a year per OT
	Grimmond + Reiner 2012^102^	Reusable vs. disposable: sharps containers	RSC = hospital reduced its annual emissions by 127 MTCO_2_eq		RSC = diverted 30.9 tons of plastic and 5.0 tons of cardboard from landfill
	Deshpande 2021^100^	Other: Familiar scrub nurse and surgeon vs unfamiliar			Greater surgeon-scrub familiarity was associated with lower risk of waste generation

### Australia

Six studies^74–79^ used LCA calculations to estimate the change in GHG emissions in relation to reusing, reducing, and refusing practices in Australia. Switching to reusable items instead of single-use items might be ineffective or even more environmentally damaging due to high water and energy consumption during cleaning and sterilisation, and due to Australia’s reliance on coal as an energy source. For instance, CO_2_e emissions were estimated to increase by 0.9% per operation when switching to reusable ureteroscope^75^, by 15% for reusable anaesthetic trays^79^, and 66.39% for and Central Venous Catheter insertion kits^76^. It estimated the environmental impact of switching to reusable anaesthetic equipment in the UK and the USA, and showed reductions of 84 and 48%, respectively. However, the same intervention would result in a 9% increase of CO_2_e emissions in Australia^79^.

For similar reasons as above, in the ‘refuse’ category, changing to spinal anaesthesia or combined anaesthesia would have comparable emissions^78^ because of the longer duration of anaesthesia for spinal and combined which contributes to CO_2_e, particularly electricity for the air warmer. Consequently, it appears that a ‘reduce’ strategy maybe the most impactful as it would avoid the above pitfalls. McGain *et al*.^80^ revealed that reducing energy consumption by switching off steam sterilisers when idle would decrease electricity by 26% and water by 13%.

### The European Union (EU)

Six studied^81–86^ estimated the environmental impact of changing practices in relation to ‘reuse’ and ‘reduce’ practices in the EU. No general statement for or against disposable surgical instruments can be made, and these should be decided on a case-by-case basis. This is largely because facilities employ different sterilisation processes and may outsource them. Since sterilisation is responsible for up to 90% of the GHGe of the reusable instruments, this can be the main factor in deciding which is more sustainable^84^. Other factors such as production sites, and disposal and recycling facilities add to the complexity of deciding which system is more sustainable.

Nevertheless, a ‘reuse’ strategy could be financially and environmentally beneficial. For instance, according to LCA calculations, switching to reusable laparoscopic instruments^81^, reusable laparotomy pads, and reusable scissors can result in financial and environmental savings. Conversely, Leiden *et al*.^84^ revealed that disposable spinal fusion surgery sets have lower environmental impact compared to reusable sets, due to local steam sterilisation processes and the big size of the reusable surgery instrument set compared to gamma radiation sterilisation process and the modular nature of disposable sets. It is noteworthy to mention that the production site of the reusable set in the study was in the USA, while the production site of the disposable set was Switzerland, which highlights the complexity of LCA calculations.

Similarly to Australia, a ‘reduce’ strategy would guarantee environmental benefits. Traversari *et al*.^86^ estimated a 70% of energy consumption from surgery could be saved by switching of the ventilation systems during prolonged inactivity (during the night and weekend) with no effect on air quality, saving 2% of the total electric energy consumption of the hospital^85^.

### The United Kingdom (UK)

Nine comparison studies^87–95^ were conducted in the UK and addressed ‘reuse’, ‘refuse’, and ‘reduce’. Firstly, reducing the ventilation rate of operating rooms from 30 to 6 air changes per hour can reduce the annual energy cost of OTs by 70%^92^. In the ‘reuse’ category, as is the case in the EU, no general statement for or against reusable surgical instruments can be made. The carbon footprint per instrument was found to be lower for single-use tray wraps (13 g CO_2_e) compared to reusable aluminium containers (25 g CO_2_e) and flexible pouches (44 g CO_2_e)^94^. However, a 22% reduction in CO_2_e could be achieved using reusable instrument sets for skin surgery^90^. Additionally, there appears to be a dissonance between the financial cost and the environmental cost of instruments. The carbon footprint of using hybrid (reusable and single-use) devices in laparoscopic cholecystectomy was 24% of the level associated with single-use devices. However, life cycle cost of hybrid devices was 46% of that of single-use equivalents (£131 vs. £282) per operation^93^.

In the refuse category, the environmental impact of decontaminating and packaging instruments was 2–3 times higher when instruments were individually wrapped instead of included in sets^94^. Scrubbing with alcohol can save 18.5 l of water per staff member^91^, and using ‘knee on’ taps instead of ‘elbow on’ taps can save 5.7 l of water per staff member^95^. Regarding the type of anaesthesia, practices such as using an oxygen/air mix at the lowest flow rate with vapour capture technology could reduce the environmental impact of inhaled anaesthetic gases to similar levels as intravenous propofol^89^. Lastly, in cases requiring 10 or fewer instruments, opting for individually wrapped items was deemed more favourable than opening another set that has the item^94^.

### USA

Eighteen comparison studies were identified in the USA^54,72,96–110^. All studies in the ‘reuse’ category demonstrated positive impacts. For example, reusable steel laryngoscope handles, subjected to high-level disinfection, produce 25 times fewer GHG emissions than single-use laryngoscope handles and are associated with financial savings^104^. Overall, switching to reusable materials can result in substantial reductions in the environmental impact, for example laryngeal mask airways (35%)^111^, reusable isolation gowns^108^ (30%), reusable surgical gowns (66–84% in GHGe^109^ and 65% in waste^99^), and reusable sharps containers (65%^103^–83.5%^102^).

In the refuse category, performing minimally-invasive operations (robotic and laparoscopic) produces more GHGe than open surgery due higher energy requirements and waste generation^105,110^. However, the LCA calculations do not include the postoperative hospital stay and care, which is thought to be less in the minimally invasive approach. Additionally, performing procedures such as Carpal Tunnel Release in clinics, rather than in OTs, can safely reduce safely reduce waste by 86% and cost by 85%^98^. Finally, Thiel *et al*.^106^ concluded that the carbon footprint of a hysterectomy operation could be reduced up to 83%, through optimising the instrument tray via use of minimal materials and maximum reuse (49%), switching anaesthesia to intravenous anaesthesia with propofol or similar agents (28%), and using renewable energy (6%).

## Discussion

This umbrella review examines the interventions aimed at reducing the environmental footprint of operating theatres using the BCW and TBL frameworks. It is evident that policymakers must scrutinise the intervention within the context of their specific geographic locations and institutional culture; and that regional variations in healthcare practices, waste management systems, and environmental regulations necessitate a tailored approach. Reduction of energy consumption and surgical waste emerged as one of the most impactful strategies, yielding environmental and financial benefits regardless of geographic factors. This strategy, though challenging to quantify, relied predominantly on staff education^46,48–50,55–58^ and environmental restrictions^52–58^.

A recent systematic review by Almukhtar *et al*.^31^ revealed that ‘knowledge’ (e.g. awareness of sustainable practices) and ‘environmental context and resources’ (e.g. personnel shortage, workload, and inadequate recycling facilities) were the most commonly identified barriers to the adoption of environmentally sustainable practices in OTs. This is consistent with the findings from this review, which identified that ‘staff education’ and ‘environmental restrictions’ are the predominantly used IFs in OTs. Targeted educational programmes highlight the cost implications of different equipment choices, instil a sense of responsibility, and drive behaviour change among healthcare professionals. Education regarding the environmental impact of consumables and equipment encourages users to consider environmental cost, which results in more sustainable choices and minimising unnecessary waste.

Simultaneously, environmental restructuring involves practical changes in the physical environment and operational processes. By combining the awareness generated through education with the tangible changes brought about by environmental restructuring, a synergistic effect can be achieved. Initiatives such as waste segregation^52,55,57,58^, recycling programs^51^, and streamlining packages^54^ have been shown to reduce surgical waste in North America, Europe, and Asia. This dual approach not only builds a lasting sense of responsibility, but it also provides the necessary infrastructure to support and reinforce sustainable practices, ultimately contributing to the reduction of waste in OTs. However, behavioural interventions often require ongoing support, reminders, and a conducive environment for long-term efficacy. The success of such interventions may depend on the degree of engagement and motivation among staff members, making sustained behaviour change a complex and multifaceted challenge as described by McCarthy *et al*.^46^.

The presence of a dedicated sustainability committee has been shown as a key facilitator in influencing behavioural change in surgery^47,68,70,71^. Challenging behaviours, especially those pertaining to perceived safety (such as switching to reusables^47^ and the reprocessing of single-use surgical devices^47,68^) can be successfully achieved and sustained with the help of dedicated ‘champions’. The approach can effectively communicate the safety, effectiveness, and reliability of sustainable practices. Moreover, a committee composed of different staff members can serve as a platform for collaborative discussions, enabling the exchange of insights and iterative strategies to achieve sustainable impact and a behavioural shift among diverse stakeholders^70,71^.

Recycling initiatives, predominantly implemented through interventions involving environmental structuring such as labelling^51^, using coloured bins^63^, waste segregation^64^, and expanding existing recycling initiatives^43,61^ played a pivotal role in reducing the environmental impact by promoting the reuse of materials and minimising waste generation. However, McGain *et al*.^79^ serves as a crucial focal point in the discussion on recycling practices in healthcare waste management. The comparative analysis across Australia, the UK/Europe, and the USA reveals that the conversion of single-use to reusable anaesthetic equipment in Australian hospitals would lead to a surprising 9% increase in emissions. In contrast, hospitals in the UK and the USA would experience significant reductions of 84 and 48%, respectively. This stark contrast showcases the crucial role of local industrial and infrastructural contexts which determine the environmental impact of recycling initiatives. Additionally, factoring the financial aspect into the decision-making further complicates the matter. The same study estimated a 46% decrease in the annual costs of a hospital in Australia following the conversion to reusable anaesthetic equipment. Furthermore, it is estimated that a small hospital in the UK running 10 elective operating theatres can make annual savings of £83 000 by switching to reusable personal protective equipment^112^. This economic perspective adds complexity to the discussion, suggesting that while recycling practices may or may not yield environmental benefits in some regions, the financial considerations may prevail.

While interventions with a single IF have demonstrated their effectiveness, they may lack the synergies that emerge when multiple elements are addressed concurrently in multifaceted-approach interventions. This review demonstrates that intervention packages incorporating multiple IFs have proven to be highly effective in reducing the environmental impact of surgery by addressing multiple barriers and integrating multiple strategies^51,55–58,68,70,71^. For instance, multifaceted-approach interventions incorporating education, environmental restructuring, and enablement, have achieved substantial waste reduction, improved recycling, and energy efficiency^70,71^. These packages provide a holistic response by acknowledging the diversity of processes and stakeholders involved in healthcare settings. The synergy between different IFs such as education and environmental restructuring creates a reinforcing effect, enhancing overall effectiveness. Moreover, beyond behaviour change, intervention packages contribute to cultivating a sustainable culture within healthcare fostering enduring environmental consciousness among professionals and aligning organisational norms with eco-friendly practices.

Currently, comparing the outcomes of interventions, even when utilising a well-recognised framework such as TBL, is difficult and potentially misleading; highlighting the pressing need for a tool that can compare and integrate diverse outcomes and contextual factors. The importance of context-specific considerations when implementing interventions cannot be understated. As highlighted by MacNeil *et al*.^73^ and demonstrated in LCA studies, there is no one-size-fits-all solution. Factors such as energy sources, waste management systems, and local conditions significantly impact the environmental outcomes of interventions. However, a ‘reduce’ strategy consistently emerged as a reliable and successful approach in healthcare sustainability across nations and can serve as a foundational principle when designing sustainability interventions.

Our scoping review revealed a number of systematic reviews looking into sustainability interventions in surgery, however, these focussed on particular specialities^16^, surgical procedures^16^, or outcomes^17^. Moreover, some of these interventions were missed in the reviews that aimed to have a broad scope^14,18,19^ leading to notable omissions (as illustrated in Fig. 2). As such, we opted to perform an umbrella review of the published reviews to examine the evidence from multiple research syntheses, compare and contrast, and provide a high-quality overall assessment of the literature in relation to sustainability in operating theatres^113,114^. This would have not been possible to achieve by searching for papers as the search strategy, when attempted, had to be extremely broad in order to capture all of these studies. Consequently, it yielded an extensive number of irrelevant articles increasing the chances of human error^115^. As a result, and after consulting with a medical librarian, we decided to conduct an umbrella review. It is a recommended approach when there is a large number of reviews on an important topic. It enables the findings of separate reviews to be compared and contrasted, providing decision makers with the evidence they need^20^.

The review is constrained by the limitations inherent in the existing body of research. For instance, methodological challenges may impact the precision and extent of the reported environmental outcomes in studies. Additionally, some reviews included older studies. For instance, of the 43 comparison studies 3 were published before the year 2010. When scrutinised, it was evident that the older studies reported similar findings to more recent studies. For instance, Kummerer *et al*. (1996)^83^ found that disposable laparotomy pads have higher emissions. These were similar to Vozolla *et al*. (2018^108^ and 2020^109^) who demonstrated that disposable gowns have higher emissions. Although the country-specific findings from Kummerer *et al*. can be explained by the findings from Leiden *et al*. (2020)^84^, comparing the outcome of different interventions can be misleading. Furthermore, different health systems have different environmental practices and policies. While some systems are no longer used in some countries, other countries might still be using these systems. Nonetheless, this review is unique in its approach and findings. It is comprehensive and distinguishes between tested interventions and estimates, and applies rigorous behavioural and environmental frameworks to provide reliable, robust and insightful evidence, and recommendations for sustainable practices in surgery.

## Conclusion and recommendations

Policymakers must scrutinise interventions within the context of their local specific healthcare practices, energy sources, waste management systems, and environmental regulations. Of the five Rs, the ‘reduce’ strategy is potentially the most impactful and was achieved predominantly through ‘staff education’ and ‘environmental restructuring’. Multifaceted-approach interventions to changing behaviour have proven to be highly effective and the presence of a dedicated sustainability committee was found to be a key facilitator in influencing behavioural change in surgery. Comparing the environmental outcomes of different interventions is currently impossible and potentially misleading, even when utilising a well-recognised framework such as the TBL. There is a pressing need for a tool that can integrate and synthesise diverse outcomes and metrics from different interventions to enable useful comparisons.

## Ethical approval

Ethical approval not needed – systematic review.

## Consent

Not applicable.

## Source of funding

This work was supported by the Medical Research Council grants [MR/X011720/1, MR/X502959/1], and a National Institute for Health and Care Research (NIHR) fellowship.

## Author contribution

A.A., M.B., J.W.B., D.L., T.P., P.D., and G.J.: contributed to conception and design of the study; A.A., C.B., and M.B.: contributed to acquisition and analysis of data; A.A., C.B., M.B., G.J., P.D., and T.P.: contributed to interpretation of data; C.B., A.A., T.P., P.D., and G.J.: wrote the manuscript. All authors contributed to manuscript revision, read, and approved the submitted version.

## Conflicts of interest disclosure

The authors declare that the research was conducted in the absence of any commercial or financial relationships that could be construed as a potential conflict of interest.

## Research registration unique identifying number (UIN)

Registered on PROSPERO: CRD42024501755.

## Guarantor

Aws Almukhtar, Carys Batcup, Miranda Bowland, Talya Porat, Pelin Demirel, and Gaby Judah.

## Data availability statement

The data used in this review are extracted from publicly available studies. The Behaviour Change Wheel is freely accessible and available. Tables are included in the body of the manuscript. Template data collection forms, data extracted from included studies, and data used for analysis are available upon request.

## Provenance and peer review

Not commissioned, externally peer-review.

## Supplementary Material
