# Supplementary material for: Interventions to achieve environmentally sustainable operating theatres: an umbrella systematic review using the behaviour change wheel
Source: Int J Surg. 2024 Aug 2;110(11):7245–67. doi: 10.1097/JS9.0000000000001951 (PMC11573083; doi:10.1097/JS9.0000000000001951)
Supplement: Supplementary file 2 [file js9-110-7245-s002.docx]

**Supplementary material:**

**TABLE 1: Summary of search terms used**

| **Search Filter** | **MeSH terms** | **Non-MeSH terms** | |
| --- | --- | --- | --- |
|  |  | **Sustainability** | **Surgery** |
| ((((systematic* adj2 review) or Cochrane review* or systemic) adj2 review*) or scoping review or scoping literature review or mapping review or Umbrella review* or review of reviews or overview of reviews or meta-review or integrative review or integrated review or integrative overview or meta-synthesis or metasynthesis or quantitative review or quantitative synthesis or research synthesis or meta-ethnography or Systematic literature search or Systematic literature research or meta-analyses or metaanalyses or metaanalysis or meta-analysis or meta-analytic review or meta-analytical review or meta-analysis). | Meta-analysis  systematic review  climate change  Conservation of Natural Resources  Carbon Footprint  Greenhouse Gas  operating room | recycle* adj2 sustainab*  environment* adj2 sustainab*  climate change  lifecycle  carbon footprint*  recycle* footprint  environment* pollution* greenhouse  recycle* | Surge*  Surgical  Operation* adj2 theatre*  Operat* adj2 Theatre*  Operat* adj2 room |

*MeSH, Medical Subject Heading;*

*Truncation symbols (*),* *used as a substitute for any string of zero or more characters at the end of a word.*

*Wildcard symbol (?) can be used as a substitute for one character or none;*

*Adj2, adjacency searching for both terms and up to one word in between them*

*We used “AND” conjunction between the different three categories using MeSH and Non-MeSH terms: “Barriers and facilitators” terms AND “Sustainability” terms AND “Surgery” terms*

**Table 2: Inclusion and exclusion criteria for the search**

| **Inclusion** | **Exclusion** |
| --- | --- |
| Population: patients or staff | Population: non-surgical staff or patients, Age of participants less than 18 years, Studies with less than 10 participants. |
| Intervention or a comparison study regarding sustainability in surgery | - |
| Setting: surgery | Setting: non-surgical specialty |
| Study designs: any | Commentaries, Editorials, Letters, Abstract only published, Conference posters and Case report |
| Study language: English only | Language other than English |

**TABLE 3: JBI quality assessment**

| Author (date) | Is the review question clearly and explicitly stated? | Were the inclusion criteria appropriate for the review question? | Was the search strategy appropriate? | Were the sources and resources used to search for studies adequate? | Were the criteria for appraising studies appropriate? | Was critical appraisal conducted by two or more reviewers independently? | Were the methods used to combine studies appropriate? | Was the likelihood of publication bias assessed? | Were recommendations for policy and/or practice supported by the reported data? | Were the specific directives for new research appropriate? |
| --- | --- | --- | --- | --- | --- | --- | --- | --- | --- | --- |
| Allwright & Abbott (2021) | Met | Met | Met | Not Met | Not Met | Not Met | Unclear | Not Applicable | Unclear | Met |
| Bhangu et al. (2023) | Met | Met | Met | Met | Not Met | Not Met | Met | Not Applicable | Met | Met |
| Bolten et al. (2022) | Met | Met | Met | Met | Not Met | Not Met | Met | Not Applicable | Met | Met |
| Bravo et al. (2022) | Met | Met | Met | Met | Not Met | Not Met | Met | Not Applicable | Met | Met |
| Drew et al. (2021) | Met | Met | Met | Met | Met | Met | Met | Not Applicable | Met | Met |
| Guetter et al. (2018) | Met | Met | Met | Met | Not Met | Not Met | Met | Not Applicable | Met | Met |
| Kwakye et al. (2011) | Met | Met | Met | Not Met | Not Met | Not Met | Met | Not Applicable | Met | Met |
| Lam et al. (2023) | Met | Met | Met | Met | Met | Met | Met | Not Applicable | Met | Met |
| Mubarak et al. (2023) | Met | Met | Met | Met | Not Met | Not Met | Unclear | Not Applicable | Unclear | Met |
| Perry et al. (2022) | Met | Met | Met | Met | Met | Met | Met | Not Applicable | Met | Met |
| Pradere et al. (2022) | Met | Met | Met | Met | Not Met | Not Met | Met | Not Applicable | Met | Met |
| Reynier et al. (2021) | Met | Met | Met | Not Met | Not Met | Not Met | Met | Not Applicable | Met | Met |
| Rizan et al. (2020) | Met | Met | Met | Met | Met | Met | Met | Not Applicable | Met | Met |
| Shoham et al. (2022) | Met | Met | Met | Met | Not Met | Not Met | Met | Not Applicable | Met | Met |
| Shum et al. (2022) | Met | Met | Met | Met | Not Met | Not Met | Met | Not Applicable | Met | Met |
| Sullivan et al. (2023) | Met | Met | Met | Met | Not Met | Not Met | Met | Not Applicable | Met | Met |

Table 4: reviews characteristics data extraction sheet template

| Author (date) | Country | Study design | Aim of review | Dates | Search strategy | Number of studies included in review | Number of studies with: environmental focus, intervention/ comparison, not reviews or reports, are published, not conference abstracts, focused on surgery/OT, not just an LCA (must be comparing at least two options) | Main findings | Conclusions |
| --- | --- | --- | --- | --- | --- | --- | --- | --- | --- |

Table 5: study characteristics data extraction sheet template

| Review/s | Study author and year | Country | Intervention focus | 5R category | BCW | | Intervention details | Outcomes | | | |
| --- | --- | --- | --- | --- | --- | --- | --- | --- | --- | --- | --- |
|  |  |  |  |  | Intervention Function | Policy category |  | Waste | Emissions or CO2e | Financial | Other |
